# Manipulating Memory Associations Minimizes Avoidance Behavior

**DOI:** 10.3389/fnbeh.2021.746161

**Published:** 2021-11-03

**Authors:** Jianqin Wang, Tom Smeets, Henry Otgaar, Mark L. Howe

**Affiliations:** ^1^Laboratory of Social Psychology and Behavioral Science, Department of Psychology, Fudan University, Shanghai, China; ^2^Department of Medical and Clinical Psychology, Tilburg University, Tilburg, Netherlands; ^3^Faculty of Law, Katholieke Universiteit Leuven, Leuven, Belgium; ^4^Faculty of Psychology and Neuroscience, Maastricht University, Maastricht, Netherlands; ^5^Department of Psychology, City University of London, London, United Kingdom

**Keywords:** memory, sensory preconditioning, false feedback, avoidance, subjective fear ratings

## Abstract

Memories of the past can guide humans to avoid harm. The logical consequence of this is if memories are changed, avoidance behavior should be affected. More than 80 years of false memory research has shown that people’s memory can be re-constructed or distorted by receiving suggestive false feedback. The current study examined whether manipulating people’s memories of learned associations would impact fear related behavior. A modified sensory preconditioning paradigm of fear learning was used. Critically, in a memory test after fear learning, participants received verbal false feedback to change their memory associations. After receiving the false feedback, participants’ beliefs and memories ratings for learned associations decreased significantly compared to the no feedback condition. Furthermore, in the false feedback condition, participants no longer showed avoidance to fear conditioned stimuli and relevant subjective fear ratings dropped significantly. Our results suggest that manipulating memory associations might minimize avoidance behavior in fear conditioning. These data also highlight the role of memory in higher order conditioning.

## Manipulating Memory Associations Mimimizes Avoidance Behavior

In the early 1930s, one of Pavlov’s dogs demonstrated the sensory preconditioning effect (see Kimmel, 1977). A whistle and a light were paired together several times, after which the dog was conditioned to flex its limb (using electric shock) upon presentation of the light. This resulted in the whistle also eliciting limb flexion, even though the whistle had never brought the dog harm (see also Brogden, 1939). Sensory preconditioning illustrates the generalization of fear responses from conditioned stimuli to neutral stimuli, which is a common symptom in anxiety disorders such as specific phobia, generalized anxiety disorder, and post-traumatic stress disorder (Dymond et al., 2015). Hence, it is crucial to understand the underlying mechanisms of the sensory preconditioning effect, such as why such fear generalization happens and how it can be interrupted.

In Pavlov’s sensory preconditioning experiment, the dog obviously formed the “whistle-light” association as well as the “light-shock” association, and somehow integrated these two memory associations to guide its reaction toward the whistle. This reaction implies that memory plays a central role in sensory preconditioning learning because if either of the memory associations was not properly remembered, the dog should not fear the whistle. Surprisingly, the question how memory plays a role in sensory preconditioning has long been neglected (e.g., Shohamy and Daw, 2015), probably due to the fact that animal subjects were mostly used in sensory preconditioning studies and it is not possible to ask animals what they remember about their fear experiences.

Recently, by testing human participants in conditioning paradigms, researchers have discovered the close link between explicit episodic memory and Pavlovian conditioning. On the one hand, fear conditioning can selectively prioritize fear related memories in long-term episodic memory (Dunsmoor and Kroes, 2019). For example, using a trial-unique fear conditioning paradigm, researchers found that people remembered the fear conditioned stimuli (CS +) better compared to the non-conditioned stimuli (CS-), and even memories of CS + related stimuli that were not conditioned got strengthened (Dunsmoor et al., 2015). On the other hand, memory has been found to play a role in various Pavlovian conditioning paradigms. Wimmer and Shohamy (2012) examined the neural mechanisms underlying human sensory preconditioning and observed that the preconditioning effect was predicted by activity in the hippocampus, where associated memories are usually formed. Other studies have found that forgetting or priming a specific memory can impact conditioned decision making (Murty et al., 2016; Bornstein et al., 2017). However, these studies were limited in using a reward learning task but did not examine the role of memory in fear conditioning. More recently, Bernstein et al. (2021) tested memory abilities of patients with anxiety disorders and found that poor mnemonic discrimination predicted overgeneralization of fear.

Taken together, the above studies suggest the possibly important role of memory in guiding (pre)conditioned behavior. Based on this observation, we wondered if fear related memories were to be manipulated, would fear conditioned behavior be impacted as well? It has been well established that human memory is a highly adaptive and constructive system where its elements can be easily manipulated *via* false feedback (Loftus, 2005; Frenda et al., 2011; Schacter, 2012). A classical study showed that participants misremembered seeing a “stop” sign after they received a verbal misleading information while in fact there was a yield sign (Loftus, 1975). More recent studies showed that encoded memories could be undermined or weakened after receiving false (verbal) feedback (Mazzoni et al., 2014; Otgaar et al., 2014; Wang et al., 2017, 2019; Li et al., 2020). For example, after participants performed actions such as clapping their hands in front of a video camera, their memories of the performed actions were tested a few days later, and false feedback was provided telling participants that their memories were wrong and some actions were never performed (Mazzoni et al., 2014). Participants’ beliefs in their memories dropped significantly and some recollective aspects of their memories such as spatial and temporal clarity became weaker after receiving false feedback.

In a recent study, false feedback was provided regarding learned associations in a reward preconditioning task, and participants’ learned memory associations were successfully undermined (Wang et al., 2019). In the study, participants learned that a picture (S1 +) was always paired with a patterned circle (S2 +) and the S2 + stimulus was later rewarded with money (US). Participants normally preferred the S1 + stimulus because the monetary value could be transferred to S1 + *via* S2 + in the memory network. However, after telling participants that their memories were wrong (e.g., the S1 + was not paired with S2 +), their associative memories between S1 + and S2 + were weakened significantly, leading to no preference to the S1 + any more. According to the spreading activation account of memory (Anderson, 1983; Roediger et al., 2001; Howe et al., 2009), S1-S2 association as well as S2-US association could be established in the memory network after learning. Attenuating the S1-S2 memory association thus could have interrupted the value transfer from S2 to S1 while the value transfer from US to S2 remained intact. This study again demonstrated the malleability of memory as well as the crucial role of memory in sensory preconditioning. Based on the reviewed results, we reasoned that fear related behavior could be modulated by providing false feedback to fear related memory associations.

To our knowledge, no research has been conducted concerning the manipulation of fear related memories and its consequences on fear conditioned behavior. By using a modified sensory preconditioning paradigm, the current study aimed to investigate the impact of manipulating memory associations on fear avoidance behavior and subjective fear ratings. Specifically, participants first learned associations between S1 + pictures and S2 + circles and then learned that S2 + stimuli led to noise. In a memory test later, participants were falsely told that the S1 + picture was not paired with the S2 + circle, but was associated with another non-conditioned circle. Based on the spreading activation theories (Anderson, 1983; Roediger et al., 2001; Howe et al., 2009), participants would be conditioned to form “picture—circle—noise” associations in the memory network. Thus fear of noise could be spread to the preconditioned picture *via* the conditioned circle. By providing false feedback to weaken the “picture—circle” association, the transfer of fear to the picture should be reduced. Therefore we expected that fear avoidance and subjective fear of S1 + pictures should be impacted by receiving false feedback.

## Methods

### Participants

Before recruiting participants, we used G^∗^Power 3.1 (Faul et al., 2007) to calculate the required sample size. With an estimated medium effect (*d* = 0.4) based on previous research (Wang et al., 2019), an *a priori* power analysis revealed that 52 participants were required to achieve a power of 0.80 (selecting *t test, matched pairs* in G. Power). Fifty-two students from Maastricht University, Netherlands, participated in our study either for course credits or a financial reward of €7.5. The sample consisted of 16 males and 36 females, with age ranging from 18 to 57 years old (*M_age_* = 23.56, SD = 6.9). The study was approved by the ethical committee of the Faculty of Psychology and Neuroscience, Maastricht University. This study was pre-registered on the Open Science Framework^1^.

### Design and Procedure

The study adhered to a within-subject design in which we provided either false feedback or no feedback in the memory test in order to manipulate memory associations. During the memory test, half of the associations received false feedback to break their established associations and the other half received no feedback (i.e., the control condition). The procedure basically followed the same steps as in previous sensory preconditioning research but with a memory feedback phase inserted before measuring fear (e.g., Wimmer and Shohamy, 2012; Wang et al., 2019). A loud blust of white noise served as the unconditioned stimulus (US) as a large body of research has validated the effectiveness of noise to induce conditioned fear responses (see Mueller et al., 2014; Sperl et al., 2016; Lonsdorf et al., 2017). The US intensity (75–105 dB, with 5 dB intervals) was calibrated for each participant before the experiment so that the noise as was perceived as unpleasant, but not painful by each participant. For instance, participants heard the lowest noise first and each time the noise was increased by 5 dB until it reached the participant’s threshold. The experiment contained the following four phases.

#### Preconditioning Phase 1: Association Phase

**FIGURE 1:**
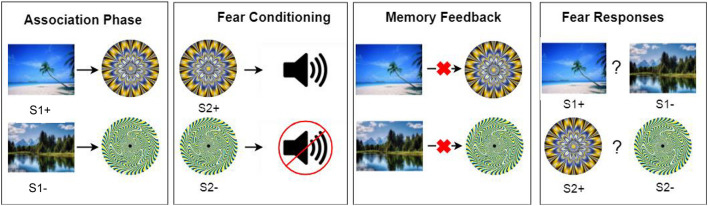
A brief illustration of the procedure. Here illustrates one of the four picture categories (i.e., the scenery pictures). E-prime was used to present all stimuli. All S1 and S2 materials were generated from Wang et al. (2019).

As Figure 1 shows, in the first phase, neutral pictures were paired with neutral patterned circles. Participants were only instructed to view some pictures on screen but were not explicitly told to memorize associations. A picture always appeared before a particular patterned circle. Each stimulus was presented for 1.5 s. The interval between the picture and the circle was 1 s and the interval between separate pairs was 3.5 s. Each pair was presented ten times, in randomized order. There were four categories of pictures (scene, furniture, body part or vehicle) and each category contained two pictures, a S1 + picture that was paired with a later fear conditioned circle and a S1- picture that was paired with a non-conditioned circle. Materials were counterbalanced in that each picture had equal chance to be a S1 + or S1- picture. Four filler pairs were also presented so that there were not too few items tested in the upcoming memory test and fear measurement phase. After all pairs were presented, participants rated their anxiety, arousal, pleasantness and liking for each stimulus on a 1–7 Likert scale to measure their baseline subjective affect ratings (Sperl et al., 2016; Lonsdorf et al., 2017).

#### Preconditioning Phase 2: Fear Conditioning Phase

During this phase, half of the circles (S2 +) that had been presented in the association phase were followed by a loud burst of white noise (US). The other half of the circles, labeled as S2- stimuli, were never paired with the aversive noise. Noise was administered *via* over-ear headphones. Each S2 + stimulus was conditioned 16 times, with 100% contingency rate while each S2- stimulus was presented 16 times but not conditioned (Wimmer and Shohamy, 2012).

#### Memory Feedback Phase

After the preconditioning phase, participants put down the headphones to receive instructions from the experimenter and to avoid any potential learning in the memory test. They completed an incidental memory test for learned associations in the first phase. Participants had to recognize which circle was paired with a particular S1 picture (two choices were provided: a correct one and a wrong one). Four associations (two S1 + and two S1- associations) were provided with false feedback after their recognition to undermine their memories. The computer program falsely indicated that the other (actually incorrect) association was the correct answer. Additionally, the experimenter verbally informed the participant that their memory was wrong and that the experimenter had clearly seen that the image was actually paired with the other, incorrect circle. Four other associations and four filler picture pairs received no feedback (i.e., no memory manipulation). After each recognition, participants were asked to rate their recollection (“Do you actually remember that the two items were paired together?”) and belief (“Do you believe that the two items were paired together?”) for the original memory association on an 8-point scale (1 = no memory or belief at all, 8 = complete memory or belief; Scoboria et al., 2014).

#### Fear Response Measurement Phase

Finally, participants went through the fear response phase to measure their avoidance behavior. For each trial, two pictures or two circles appeared left and right on screen. Participants were asked to choose a picture to avoid noise by pressing the F (left) or J (right) button, and choosing a wrong picture would bring a noise lasting 2 s. Such operant responses have been used in previous research to measure the preconditioning effect (Wimmer and Shohamy, 2012), which mimicked operant fear measurement in rodents (e.g., choosing between two chambers to avoid shock; Krypotos et al., 2015). Headphones were put up again so that they could receive the noise. Each trial consisted of a S1 + picture and a S1- picture from the same category (e.g., beach vs. lake or leg vs. arm). The S2 + and S2- circles were presented in another trial to assess fear learning. The same two stimuli were presented for four times, with each stimulus randomly appeared on the left or right side. To avoid re-learning in the fear measurement phase, noise was not administered immediately after each trial, but participants were told that noise would be accumulated if they made the wrong choice and they would receive a certain amount of noise in the end of each block. S1 pairs and S2 pairs were intermixed in each block. There were a practice block and two official blocks. There were 32 critical trials in total. After all trials, participants were asked again to provide subjective affect ratings for each stimulus.

## Results

### Memory Data and Manipulation Check

Participants were asked to choose the S2 circle that they recalled was associated with a S1 picture. Memory accuracy for associations pre-false feedback [*M* = 0.60, 95%CI (0.49, 0.70)] did not differ significantly from the memory accuracy for associations in the no feedback condition [*M* = 0.67, 95%CI (0.58, 0.77)], *t* (52) = −1.16, *p* = 0.25, indicating equivalent levels of associative memories formed in the two conditions.

**FIGURE 2:**
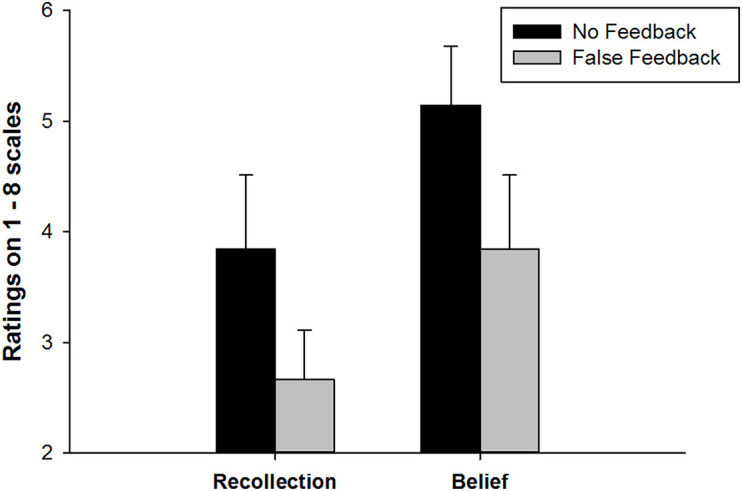
Recollection and belief ratings in False feedback and No feedback conditions. Error bars represent 95%CI.

After false feedback was provided in the memory test, participants rated their recollections and beliefs for the associations. A 2 Memory component (Recollection vs. Belief) × 2 Feedback (False vs. No) repeated measures ANOVA was conducted to examine participants’ memory ratings. As Figure 2 shows, there was a significant main effect of Feedback, *F* (1, 51) = 24.20, *p* < 0.001, partial η^2^ = 0.32, and a significant main effect of Memory component, *F* (1, 51) = 42.63, *p* < 0.001, partial η^2^ = 0.46. No interaction effect between Memory component and Feedback was found, *F* (1, 51) = 0.77, *p* = 0.38, suggesting that false feedback weakened both recollection and belief ratings of learned memory associations. Specifically, as Figure 2 shows, false feedback has lowered recollection rating at the magnitude of Cohen’s *d* = 0.71, *p* < 0.001 and lowered the belief rating with a size of Cohen’s *d* = 0.58, *p* < 0.001.

### Avoidance Behavior

#### Avoidance of S2 +

First, we needed to make sure that participants learned fear for S2 + stimuli in the fear conditioning phase in the form of avoiding S2 + later. Avoidance was operationalized as the choosing rate of a fear conditioned image in the fear response phase. Hence, the lower choosing rate of a stimulus, the more avoidance to that stimulus; and 50–50 chance of choosing a stimulus in a pair suggests no avoidance or preference. For directly fear conditioned stimuli (S2 +), participants chose overall 16.23% of the times S2 + but 83.77% of the times chose S2- to avoid noise, suggesting successful fear learning of S2 + in the form of avoiding S2 +. The mean choosing rate of S2 + in the false feedback condition [*M* = 20.19%; 95%CI (0.12, 0.28)] did not statistically differ from that in the no feedback condition [*M* = 12.26%; 95%CI (0.05, 0.19); *p* = 0.06], both of which were significantly below 50% chance level (*p*s < 0.001). These data suggest that participants learned fear of S2 + to the same extent in the two conditions.

#### Avoidance of S1 +

Next, we analyzed how fear transferred to S1 + stimuli. The key dependent variable we were interested in was the avoidance of S1 + relative to S1-, that is the choosing rate of S1 + vs. S1- stimulus in different feedback conditions. Participants again showed preconditioned fear responses in the no feedback condition. That is, they avoided choosing S1 + [*M* = 33.65%; 95%CI (0.24, 0.43)] but chose S1- more often [*M* = 66.35%; 95%CI (0.57, 0.76)], demonstrated by the significant lower choosing rate of S1 + than 50%, *t*(51) = −3.44, *p* = 0.001, Cohen’s *d* = 0.48. However, participants did not exhibit the fear preconditioning effect in the false feedback condition, that is, participants showed no avoidance to the S1 + stimuli but exhibited a choosing rate of S1 + [*M* = 43.99%; 95%CI (0.36, 0.52)] not different from chance level (50%), *t*(51) = −1.51, *p* = 0.14. Thus, false feedback decreased an absolute number of 10.34% fear avoidance choosing rate and relative 30.73% of the original fear avoidance compared to no feedback. More detailed analyses on the direct comparison between these two conditions will be discussed now.

**FIGURE 3:**
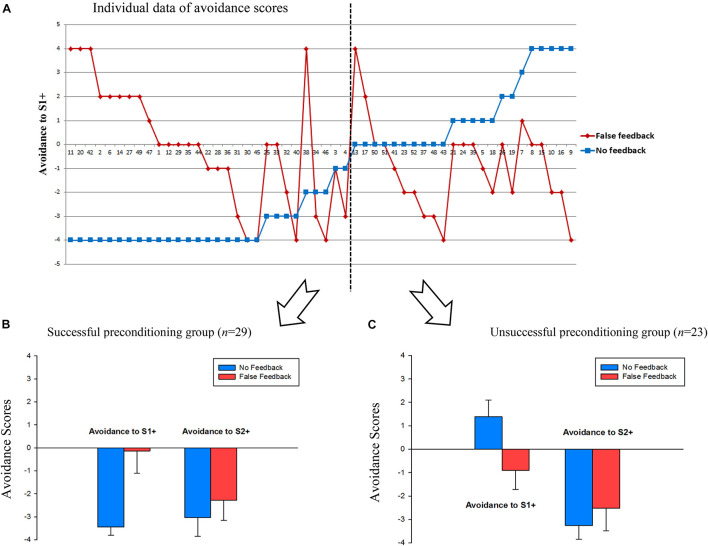
**(A)** Individual data of avoidance scores in false and no feedback conditions. Avoidance score = (Times of choosing S1 +)—(Times of choosing S1-). The smaller the value, the more avoidance to S1 +. Each number on the X-axis represents one participant. Error bars represent 95%CI. **(B)** Avoidance scores to S1 + and S2 + stimuli in the successful preconditioning group. **(C)** Avoidance scores to S1 + and S2 + stimuli in the unsuccessful preconditioning group.

To visualize participants’ avoidance behavior regarding the preconditioned stimuli (S1 +), the net avoidance score of S1 + for each participant was calculated, which was the times of choosing S1 + stimuli minus times of choosing S1- stimuli over four rounds (see Wang et al., 2019). As Figure 3 shows, a negative value indicates that participants avoided choosing S1 + stimuli over S1- stimuli; a positive value indicates participants preferred S1 + stimuli; 0 value means 50% chance level. The avoidance score ranged from −4 to 4. Figure 3A shows individual data on avoidance scores. Before analyzing the avoidance scores, it is crucial to check whether participants had been successfully preconditioned to fear S1 + stimuli in the control condition.

We found that in the control condition, there were 29 people who successfully learned the fear preconditioning (i.e., < 50% chance of choosing S1 +) and there were people (*n* = 23) who failed to learn the fear preconditioning (i.e., no avoidance or even preference of S1 +). Thus, we split participants into two groups: the successful fear preconditioning group and the unsuccessful fear preconditioning group. In the successful fear preconditioning group (*n* = 29), false feedback [*M* = −0.14, 95%CI (−1.11, 0.84)] eliminated avoidance behavior significantly relative to the no feedback condition [*M* = −3.45, 95%CI (−3.81, −3.09)], *t*(28) = 5.94, *p* < 0.001, Cohen’s *d* = 1.16; in the unsuccessful fear preconditioning group (*n* = 23), false feedback [*M* = −0.91, 95%CI (−1.73, −0.10)] still reversed the avoidance/preference behavior compared to the no feedback condition [*M* = 1.39, 95%CI (0.69, 1.09)], *t*(22) = 4.29, *p* < 0.001, Cohen’s *d* = 0.89. The individual data in Figure 3A shows that fear-avoidance behavior was impacted by false feedback at an individual level. Meanwhile, fear learning of S2 + stimuli was not impacted in both groups, i.e., participants in either feedback condition have successfully learned avoidance to S2 + (*p*s > 0.05).

### Subjective Affect Ratings

**FIGURE 4:**
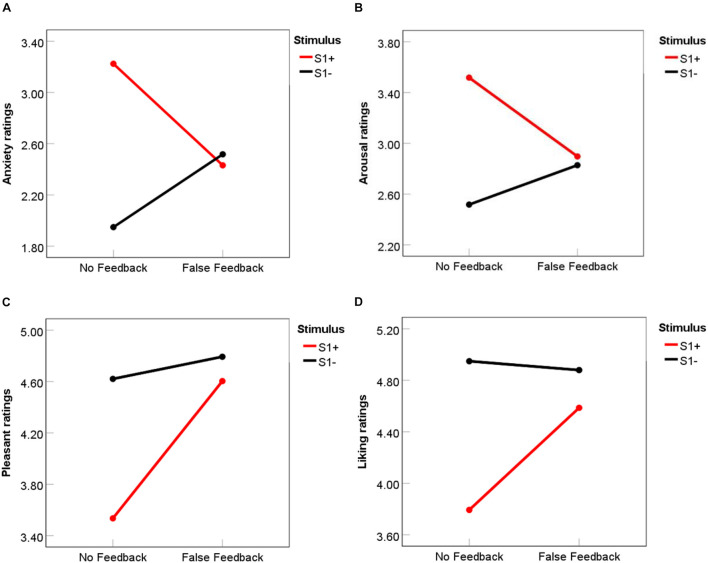
Mean ratings of anxiety **(A)**, arousal **(B)**, pleasantness **(C)**, and liking **(D)** for S1 + and S1- in false feedback and no feedback conditions. Ratings ranged from 1 to 7. For Anxiety, 1 = not anxious at all, 4 (middle point) = moderate anxiety, 7 = very anxious; For Arousal, 1 = very calm, 4 = neutral, 7 = very aroused; for Pleasantness, 1 = very unpleasant, 4 = neutral, 7 = very pleasant; for Liking, 1 = very disliked, 4 = neutral, 7 = very liked.

Before conditioning, there was no significant difference between S1 + and S1- stimuli for baseline ratings of anxiety, *t*(51) = 0.34, *p* = 0.73, arousal, *t*(51) = −0.63, *p* = 0.53, pleasantness, *t*(51) = −1.79, *p* = 0.08, or liking, *t*(51) = −0.96, *p* = 34. To examine whether false feedback affected participants’ affect ratings after the feedback phase in the successful preconditioning group, a 2 Feedback (False feedback vs. No feedback) × 2 Stimulus (S1 + vs. S1-) repeated measures ANOVA was conducted on the mean scores for each rating (anxiety, arousal, pleasantness and liking). Results showed a significant Feedback × Stimulus interaction effects on both anxiety ratings, *F* (1, 28) = 8.81, *p* = 0.006, η^2^_partial_ = 0.24, and arousal ratings, *F*(1, 28) = 4.73, *p* = 0.038, η^2^_partial_ = 0.14. As demonstrated in Figures 4A,B, in the no feedback condition, participants had significant higher anxiety and arousal ratings for S1 + than S1- stimuli, (for anxiety, *M*_difference_ = 1.28, *p* < 0.001, *d* = 0.78, for arousal, *M*_difference_ = 1.00, *p* = 0.003, *d* = 0.60). However, false feedback eliminated the discrepancies between S1 + and S1- stimuli (for anxiety, *p* = 0.78, for arousal, *p* = 0.83), making participants no longer fear S1 + stimuli.

For pleasantness ratings (Figure 4C), a similar Feedback × Stimulus interaction pattern was observed, *F* (1, 28) = 4.04, *p* = 0.05, η^2^_partial_ = 0.13. Pleasant ratings for S1 + was significantly lower than S1- in the no feedback condition, *M*_difference_ = 1.09, *p* = 0.001, *d* = 0.66, but no difference was found in the false feedback condition, *p* = 0.50. Liking ratings (Figure 4D) showed similar patterns but the interaction between feedback and stimulus did not reach significance, *F* (1, 28) = 3.27, *p* = 0.08, η^2^_partial_ = 0.11; only a main effect of stimulus was found that participants in general liked S1- more than S1 +, *F* (1, 28) = 14.92, *p* = 0.001, η^2^_partial_ = 0.35.

## Discussion

This is the first study that examined the impact of manipulating memory associations on fear avoidance behavior using a sensory preconditioning task. We found that false feedback directed at participants’ memories resulted in decreased recollection and belief ratings for their learned associations, which demonstrates the malleability of memory and is consistent with previous research (Loftus, 2005; Schacter, 2012; Wang et al., 2019). More importantly, false feedback eliminated avoidance behavior and eased participants’ subjective fear ratings relative to the control condition.

Our results support the role of explicit or episodic memory in fear learning. Episodic memory is the conscious recollection of learned experiences, including time, space or other contextual details (Tulving, 2002). The current study measured participants’ recollections of paired circles and pictures by asking them whether they actually remembered these events instead of asking them whether they knew such events (i.e., semantic), which is a common way to measure episodic memories. For a long time, episodic memory and Pavlovian fear conditioning were two isolated research fields (see a review by Dunsmoor and Kroes, 2019). The current study connects these two fields by manipulating learned associative memories in a fear preconditioning task. We found that undermining associative memories canceled avoidance behavior to the preconditioned stimuli and it reduced anxiety and arousal ratings compared to the control condition. We also measured liking and pleasantness ratings, which are opposite affects of subjective fear (Lonsdorf et al., 2017), but we only found significant changes on pleasant ratings induced by memory feedback. The reason might be that liking ratings is not directly related to fear, although it showed a similar pattern at a descriptive level, albeit not significant (*p* = 0.08). Overall, the current study points out that episodic memory might be one crucial mechanism underlying sensory preconditioning and it highlights the potential of using memory manipulation techniques to reduce fear. As we only measured avoidance behavior and subjective affect ratings, further research is needed to investigate how false feedback on memory associations may impact physiological fear responses such as skin conductance and startle responses.

The current results can be readily explained by the spreading activation account of memory (Roediger et al., 2001; Howe et al., 2009). According to this account, memory consists of mental representations of stimuli (i.e., “nodes” in a memory network) and associations between stimuli that participants have remembered from experience. For example, when a S1 + picture was paired with a S2 + circle, a “picture—circle” memory association could be encoded in the memory network; when the S2 + circle was paired with noise, a “circle—noise” could be encoded in the memory network as well. The key principle in the spreading activation account is that activation of one memory node spreads automatically to other memory nodes along the memory network. Thus, when participants saw a S1 + picture, activation was spread to a S2 + circle and then spread to noise, resulting in activation of noise when seeing a S1 + picture. As a consequence, participants should avoid S1 + pictures. In our study, false feedback attenuated the “picture—circle” memory association, so the activation spread to noise was to some extent interrupted and participants’ fear responses to S1 + pictures were reduced.

The present results also support the memory-chaining account of sensory preconditioning relative to the online-integration account (see Rizley and Rescorla, 1972; Sharpe et al., 2017; Wong et al., 2019). The online-integration account, suggests that during the S2-noise fear conditioning phase, S1-S2 associations are activated and thus S1 is associated with noise already in the fear conditioning phase (Shohamy and Daw, 2015; Wong et al., 2019). If this is the case, manipulating the S1-S2 memory associations after the fear conditioning phase should not impact the preconditioning effect because S1 has been linked with fear already during the fear conditioning phase. However, our results showed that memory manipulation *after* the fear conditioning phase minimized the preconditioning effect, which is consistent with the memory-chaining account. That is, the transfer of fear might happen at the time of testing when presence of S1 stimulus activates the S1-S2 memory association, which in turn activates the S2-noise association, so participants showed avoidance to the S1 stimulus (Rizley and Rescorla, 1972; Sharpe et al., 2017). Thus, disrupting the S1-S2 memory association can cancel the preconditioning effect. The memory-chaining account of sensory preconditioning is intriguingly similar to the spreading activation account of memory, which deserves more investigation into the role of memory in sensory preconditioning.

Previous research on the neural mechanisms of the sensory preconditioning effect showed that the medial temporal lobe (e.g., hippocampus and its surrounding regions) are responsible for the S1-S2 phase of the preconditioning effect in both rodents and humans (Wimmer and Shohamy, 2012; Holmes et al., 2018), with also the amygdala being involved in the S2-US fear conditioning phase (Gewirtz and Davis, 2000). Coincidentally, the hippocampus/parahippocampal cortex, as well as regions in the anterior prefrontal cortex and medial parietal cortex, have been found to support the encoding and retrieval of episodic memory (Squire et al., 2000; Eichenbaum and Cohen, 2001). The hippocampus is mostly involved in forming associative memories while the prefrontal cortex is related to the monitoring or evaluation of memory traces (Mitchell and Johnson, 2009). Studies found that misinformation can impact activations in the hippocampus and prefrontal cortex, resulting in possible reconstruction of memory (Okado and Stark, 2005). The present findings imply that false feedback to learned associations may involve activities in the hippocampus and prefrontal cortex, which might lead to interruption of the S1-S2 memory associations, and that the integration between these regions and the amygdala may be important in both episodic fear memory and sensory preconditioning. Future research may look at the neural structures involved in memory-based fear learning.

This study might have certain clinical implications regarding how to interrupt the overgeneralization of fear without affecting the original fear learning memories. In our study, we did not manipulate memory associations in the fear learning phase (i.e., the “circle—noise” association), but we manipulated participants’ learned associations in the preconditioning phase (i.e., the “picture—circle” association). Results showed that fear of conditioned S2 + circles remained intact but only fear of preconditioned S1 + pictures was reduced after our false feedback manipulation. This means that fear generalization to S2 + stimuli was stopped without affecting fear learning. In clinical settings, fear (over)generalization is a pathogenic marker of anxiety disorders (Lissek et al., 2010). Our study implies that cognitive methods or techniques targeting at patients’ memories might be a fruitful future direction (see Phelps and Hofmann, 2019).

To conclude, the present research showed that false feedback to participants’ learned associations minimized avoidance behavior and reduced subjective fear ratings of preconditioned stimuli. These results suggest that episodic memory might be one of the mechanisms underlying sensory preconditioning. The time has come now to investigate how principles of memory may impact fear learning and fear generalization.

## Data Availability Statement

The raw data supporting the conclusions of this article will be made available by the authors, without undue reservation.

## Ethics Statement

The studies involving human participants were reviewed and approved by Ethical Committee of the Faculty of Psychology and Neuroscience, Maastricht University. The patients/participants provided their written informed consent to participate in this study.

## Author Contributions

JW, HO, and MH conceived the idea. JW and TS designed the experiment. JW collected the data and analyzed the data. All authors contributed to the writing of the manuscript.

## Conflict of Interest

The authors declare that the research was conducted in the absence of any commercial or financial relationships that could be construed as a potential conflict of interest.

## Publisher’s Note

All claims expressed in this article are solely those of the authors and do not necessarily represent those of their affiliated organizations, or those of the publisher, the editors and the reviewers. Any product that may be evaluated in this article, or claim that may be made by its manufacturer, is not guaranteed or endorsed by the publisher.

## Footnotes

1
https://osf.io/zahu4
