# Supplementary material for: Manipulating Memory Associations Minimizes Avoidance Behavior
Source: Front Behav Neurosci. 2021 Nov 3;15:746161. doi: 10.3389/fnbeh.2021.746161 (PMC8595481; doi:10.3389/fnbeh.2021.746161)
Supplement: Supplementary file 1 [file Table_1.DOCX]

**Supplementary Materials**

Table 1

*Avoidance scores of S1 and S2 stimuli in successful and unsuccessful preconditioning groups. Means and 95% Confidence Intervals.*

|  | **Successful Preconditioning Group** | | | **Unsuccessful Preconditioning Group** | | |  |
| --- | --- | --- | --- | --- | --- | --- | --- |
|  | Avoidance S1+ | Avoidance S2+ |  | | Avoidance S1+ | Avoidance S2+ | |
| False Feedback | -0.14 [-1.11, 0.84] | -2.28 [-3.16, -1.40] |  | | -0.91 [-1.73, -0.10] | -2.52 [-3.49, -1.56] | |
| No Feedback | -3.54 [-3.81, -3.09] | -3.03 [-3.85, -2.22] |  | | 1.39 [0.69, 1.09] | -3.26 [-3.85, -2.67] | |

**Effect of false feedback in preconditioning vs. fear conditioning phases**

A 2 Feedback (False feedback vs. No feedback) × 2 Stimulus (S1+ vs. S2+) repeated measures ANOVA on avoidance was conducted for each successful and unsuccessful preconditioning group. In the successful preconditioning group, there was a statistically significant interaction between feedback and stimulus, *F*(1, 28) = 11.63, *p* = .002, η^2^_partial_ = .29. Providing false feedback did not impact avoidance of S2+ in the fear conditioning phase, *t*(28) = 1.45, *p* = .16, *d* = 0.27, but it significantly reduced avoidance of S1+, *t*(28) = 5.94, *p* < .001, *d* = 1.10. A similar interaction was found in the unsuccessful preconditioning group, *F*(1, 22) = 20.28, *p* < .001, η^2^_partial_ = .48. False feedback again did not impact avoidance of S2+ in the fear conditioning phase, *t*(22) = 1.37, *p* = .19, *d* = 0.29, but it significantly impacted avoidance of S1+, *t*(22) = 4.25, *p* < .001, *d* = 0.89.
